# Dietary patterns and their association with the components of metabolic syndrome: A cross-sectional study of adults from northeast Thailand

**DOI:** 10.12688/f1000research.15075.2

**Published:** 2019-04-03

**Authors:** Pornpimon Chupanit, Benja Muktabhant, Frank Peter Schelp

**Affiliations:** 1 Faculty of Public Health, Khon Kaen University, Khon Kaen, Thailand; 2 Research Group on Prevention and Control of Diabetes Mellitus in the Northeast of Thailand, Khon Kaen University, Khon Kaen, Thailand

**Keywords:** dietary patterns, metabolic syndrome, factor analysis, the components of metabolic syndrome, the northeast, Thailand, nutritional transition, traditional

## Abstract

**Background: **Nutritional transition influences a shift in eating behaviour that is associated with a rise in the prevalence of non-communicable diseases (NCDs). Metabolic syndrome (MetS) comprises a set of NCD risk factors. This study aimed to investigate dietary patterns and to determine the relationship between dietary patterns and MetS and its components.

**Methods: **An analytical cross-sectional study was conducted among 468 adults aged 35–60 years who were residents of a semi-urban district of one of the central provinces in the northeast of Thailand. A factor analysis identified dietary patterns based on the consumption of 21 food groups, which were assessed by using a semi-quantitative food frequency questionnaire. MetS was identified by using the harmonized criteria that were stipulated by six leading international organisations. The association between dietary patterns and MetS and its components were evaluated by multiple logistic regressions. The confounding factors adjusted in the model were age, sex, smoking status, physical activity, and medication intake.

**Results:** Two dietary patterns were identified: a traditional pattern characterised by high intakes of sticky rice and animal source foods; a mixed pattern included high intakes of white rice and a variety of food groups. The two dietary patterns did not show any association with MetS. Participants in the highest tertile of the traditional pattern was significantly related to high triglycerides (adjusted OR = 1.74, 95% CI: 1.10–2.88), in comparison to those from the lowest tertile, whereas participants in the highest tertile of the mixed pattern was inversely associated with abdominal obesity (adjusted OR= 0.49, 95% CI: 0.30–0.81) than those in the lowest tertile.

**Conclusions:** Adherence to a traditional dietary pattern among the northeast Thai adults, in the context of nutrition transition, was associated with high triglyceride levels while the mixed dietary pattern was inversely related to abdominal obesity.

## Introduction

Public health in most low- and middle-income countries is challenged, in addition to infectious diseases, by chronic or non-communicable diseases (NCDs) ^1^. This situation coincides with economic development, demographic transition, and epidemiological changes in a population, which leads to a shift in dietary behaviours and physical activity, also known as nutrition transition ^2^. The underlying dietary changes have been highlighted for Asia, including Thailand ^3^. A high consumption of red meat, processed meat, and refined sugar has increased in high- and low-income countries substantially. The changes in dietary intake are aggravated by hyper-palatable processed food high in fat, salt, or sugar ^4^. As an after-effect, NCDs such as cardiovascular diseases, especially type 2 diabetes mellitus (T2DM), have emerged as a pressing health problem because obesity and T2DM are closely related ^5^. Thailand, as a high middle-income country, was not spared from these developments and faced a rapidly increasing T2DM prevalence; the prevalence of T2DM among Thai adults has risen from 2.3% in 1991 ^6^ to 8.0% in 2015 ^7^. Thailand is divided into four major regions, and the northeast seems to have a higher prevalence of T2DM than other regions ^8^. Particular regional dietary habits might be linked to the nutritional status and subsequently, to health risks such as T2DM. Sticky or glutinous rice is a staple food and more popular in the northeastern region, whereas ordinary rice is a favourite in the other regions of Thailand ^4^. Metabolic syndrome (MetS), a cluster of metabolic abnormalities, such as impaired blood glucose, dyslipidemia, abdominal obesity, and high blood pressure, is known as a major precursor to T2DM and cardiovascular disease (CVD) ^9, 10^ A systematic review study reported that the prevalence of MetS ranged from 11% to 40% among adults in countries of the Asia-Pacific region ^11^. The fourth National Health Examination Survey in Thailand, in 2009, reported that the prevalence of MetS was 23.2% ^12^, which was somewhat higher than in other Asian countries ^13^.

In nutritional epidemiology, the analysis of dietary patterns is based on the concept of the overall food consumption and this approach was used to determine the relationship between diet and the risk of chronic diseases ^14, 15^. However, dietary patterns cannot be measured directly, thus the use of statistical methods to identify dietary patterns is necessary. Factor analysis based on intercorrelations between dietary items is the predominant method to identify dietary patterns ^16^. Several studies of dietary pattern analysis found that dietary patterns characterized by a high intake of meat, high-fat foods and refined carbohydrates were positively associated with an increase in risk of MetS and its components ^17– 19^, whereas a dietary pattern characterized by a high consumption of whole grains, fish, vegetables, and fruits was found to be protective against MetS and its components ^20, 21^. One previous investigation in Thailand identified a dietary pattern consisting of a high intake of carbohydrate in the form of sticky rice, fermented fish, chili paste and bamboo shoots that was associated with MetS and its individual components ^17^. However, the studies to determine dietary patterns in the Thai population are limited. It has remained unclear whether the traditional Thai diet or a changed pattern resulting from the nutrition transition is associated with the risk of MetS. The objective of this study was to identify dietary patterns derived from factor analysis and to determine the association between dietary patterns and MetS and its components.

## Methods

### Study population

The investigation was conducted as a cross-sectional study based on the STROBE (The Strengthening the Reporting of Observational Studies in Epidemiology) cross sectional reporting guidelines ^22^. Thai adults aged 30 to 60 years living in Nam Phong district of the Khon Kaen province were invited to take part in the study. Khon Kaen is one of the central provinces in the northeastern region of Thailand, and Nam Phong district is located close to the provincial municipality and considered a semi-urban location. A two-stage cluster random sampling was conducted to select study participants. Random villages were selected from 12 sub-districts and then each study participant from one household was recruited by using a simple random sampling method. The required sample size was estimated by using a formula for multiple logistic regression ^23^ and the desired power of 80% was used to detect a statistically significant difference. A minimum required sample size of 416 was obtained, with 15% increase to allow for potential non-responders. Therefore, the total number of samples was 478. However, only 468 samples were remained in this study due to the exclusion criteria. Subjects were excluded if they were diagnosed with cardiovascular disease, thyroid disease, cancer and other serious health conditions as well as if their total daily energy intake was <500 kcal/day or ≥5000 kcal/day. Six participants were excluded due to cardiovascular disease, thyroid disease, cancer and other serious health conditions. Another four participants were excluded because of an implausible total energy intake. The final study population was 468 participants that included 326 women and 142 men. The study protocol was approved by the Ethics Committee of Khon Kaen University for Human Research (HE552143). After informing all participants about the details of the study, they signed an informed written consent.

### Collection of demographic and health information

All participants received an appointment date to be interviewed about demographic and health information at the local primary health care center at sub-district level by trained staff. By questionnaire the following information was obtained: age; sex; smoking status; marital status; educational level; monthly income; family history of a first-degree relative with DM, hypertension (HT), and dyslipidemia; medical history and medication use for DM, HT, or dyslipidemia (Part 1 and 2 – Supplementary File 1). Physical activity (PA) was assessed by using the Global Physical Activity Questionnaire (GPAQ) ^24^. Levels of PA were classified as low, moderate, and vigorous according to the guidelines (Part 3 – Supplementary File 1). Blood pressure (BP) was measured in a sitting position using a digital sphygmomanometer (Omron Model HEM-711, Japan) by trained staffs at the local health care center after participants had rested for 5 minutes. A repeated measurement of BP was taken, and the average value was recorded.

### Anthropometric and biochemical measurement

This procedure was conducted at the local primary health care center. Anthropometric measurement was done by trained staff. Weight and height were measured by using a calibrated digital scale and a portable stadiometer while the participants were dressed in light clothing and without shoes. Waist circumference (WC) was assessed in centimeters on a horizontal plane midway between the lower end of the rib cage and the top of the iliac crest while the individual was standing. The measurements were taken twice to the nearest 0.1 cm, and the average of the values was adopted.

Blood samples were collected by a registered nurse in the morning after fasting overnight for at least 8 hours. Fasting blood glucose (FBG), triglyceride (TG), and high-density lipoprotein cholesterol (HDL-C) was determined at the laboratory of the Faculty of Medicine, Srinagarind Hospital, Khon Kaen University, by using an automatic analyser (Roche Cobas Mira-S Analyzer) in accordance with the manufacturer’s instructions for the use of reagents and operating the equipment. Fasting blood glucose was quantified by the glucose oxidase method ^25^. The levels of TG and HDL-C were measured by enzymatic methods ^26^.

### Food intake assessment

A semi-quantitative food frequency questionnaire (semi-FFQ) was used by trained assistants in a face-to-face interview at the local primary health care center to determine the participant’s food consumption (Part 4 – Supplementary File 1). The participants were asked about the frequency of consumption and the portion size of foods during the previous month. The picture of food sizes and kitchen equipment (e.g., cup, glass, spoon, table spoon, and ladle) were used for estimating the portion size of foods. The amount of food consumption was calculated into grams per day per person. The calculation of energy and nutrient intakes was done by applying the INMUCAL-N Version 2.0 computer software (provided by the Institute of Nutrition, Mahidol University based on the Thai food composition tables) ^27^. The testing of the validity and reliability of the semi-FFQ was described in detail previously ^28^. Briefly, semi-FFQ was validated with repeated 24-hour dietary recall. The Pearson’s correlation of energy and nutrient intake between two methods varied from 0.7 – 0.9.

### Dietary pattern analysis

Before the analysis of dietary patterns, a total of 104 food items in a semi-FFQ were categorized into 21 food groups based on the similarity of the nutrient profiles and local food groups specific to the northeast of Thailand for instance insects, freshwater animals (frogs, pond snails, and small shrimps), and internal organs of animals ( Table S1; Supplementary File 2). Grain and grain products are the staple food of Thai cuisine, so this group was classified into three subgroups such as white rice, sticky rice, noodles and bread. Some food items had to be listed as a single food item and could not be included into a food group because of its unique nature such as eggs, milk and yogurt, seafood, energy drinks, and alcohol.

The factor analysis through principal component analysis (PCA) was used to distinguish the dietary patterns. The principal component analysis (PCA) combines correlated food groups into factors that represent dietary patterns of the population being studied ^16, 29^. The varimax option (orthogonal rotation) was used to assemble food groups into dietary patterns. Food groups were considered a significant component of dietary pattern if they had a factor loading of > 0.3. A number of dietary patterns were retained based on eigenvalues of > 2.0 and a break point in the scree plot as well as the interpretability of each pattern. Dietary patterns were named based on the interpretation of the feature of food grouping. All participants received factor scores for each dietary pattern by summing up the intake of food groups weighted by their factor loadings. The scores of each dietary pattern were categorized into tertiles to indicate the levels of food consumption.

### Identification of the metabolic syndrome

The MetS was identified according to the harmonized criteria agreed upon from six organisations, namely the International Diabetes Federation Task Force on Epidemiology and Prevention; National Heart, Lung, and Blood Institute; American Heart Association; World Heart Federation; International Atherosclerosis Society; and International Association for the Study of Obesity ^30^. Participants with three or more of the five components were included in the group of MetS. The components of MetS are the following: (1) waist circumference ≥ 90 cm in men or ≥ 80 cm in women, (2) fasting blood glucose (FBG) ≥100 mg/dL or treated diabetes, (3) triglycerides ≥150 mg/dL, (4) low high-density lipoprotein cholesterol (HDL-C) < 40 mg/dL in men and <50 mg/dL in women, (5) systolic blood pressure (SBP) ≥130 mmHg or diastolic blood pressure (DBP) ≥85 mmHg or treated hypertension.

### Statistical methods

Categorical variables were given as numbers and percentages, and continuous variables were reported as mean ± SD. To test the differences in distribution or means of the characteristic variables of study participants, the chi-square tests for categorical variables and Student’s *t*-test for continuous variables were used. The Mann-Whitney *U* test was used if the distribution of results was not homogeneous. The magnitude of the association between dietary patterns and MetS and its components was determined by multiple logistic regression analysis, using backward elimination. The confounding factors adjusted in the model were age, sex, smoking status, physical activity, and medication intake. The overall trend of odds ratios (ORs) across tertiles of dietary pattern scores was assessed by using the Mantel-Haenszel chi-square test. The lowest or first tertile score of each dietary pattern was considered to be the reference. Statistical significance was considered at *p-*value ≤0.05. All statistical analyses were done by using STATA version 11 (Stata Corp, College Station, TX).

## Results

Characteristics of the study participants are presented in Table 1. Females, at 69.7%, were in the majority compared with 30.3% of males. The age range of women and men was not significantly different, with a mean of 49.1 ± 6.4 years. A higher proportion of females were obese (50.9%) than men (36.6%). Most of the participants were married and had finished elementary school. Men’s income exceeded that of women. Physical activity seemed to be on a vigorous level (53.2%; 68.3% in men and 46.6% in women). Only a few women smoked (1.0%), but 64.1% of the men did so. Within the group of women, 49.7% indicated that a first-degree relative had a history of diabetes, hypertension or dyslipidemia, but the proportion (36.6%) was less frequent for men. Only a minority of study participants, less than 10%, regularly took medicine for diabetes, high blood pressure, or dyslipidemia. Total energy intake and the distribution intake of carbohydrate, protein, and fat of men were significantly above that of women, but the intake of defined nutrients such as fiber, cholesterol, and sugar did not differ between the sexes.

**Table 1.** Characteristics of the study participants.

Characteristics	Total	Men	Women
Number (%)	468	142 (30.3)	326 (69.7)
Age, n (%)			
35–49 years	227 (48.5)	64 (45.1)	163 (50.0)
≥ 50 years	241 (51.5)	78 (54.9)	163 (50.0)
Mean ± SD, years	49.1 ± 6.4	49.9 ± 6.9	48.8 ± 6.3
Body mass index, n (%)			
< 25.0 kg/m ^2^	250 (53.4)	90 (63.4)	160 (49.1)
≥ 25.0 kg/m ^2^	218 (46.6)	52 (36.6)	166 (50.9)
Mean ± SD, kg/m ^2^	25.0 ± 3.9	24.0 ± 3.3	25.5 ± 4.0 *
Marital status, n (%)			
Single	14 (3.0)	5 (3.5)	9 (2.8)
Married	431 (92.1)	137 (96.5)	294 (90.2)
Widowed/Divorced	23 (4.9)	0 (0.0)	23 (7.1)
Educational level, n (%)			
Elementary school	342 (73.1)	89 (62.7)	253 (77.6)
High school	117 (25.0)	49 (34.5)	68 (20.9)
College/bachelor's degree	9 (1.9)	4 (2.8)	5 (1.5)
Monthly income, n (%)			
T _1_ (≤ 3000 bath)	178 (38.0)	35 (24.7)	143 (43.9)
T _2_ (3001–6000 bath)	138 (29.5)	48 (33.8)	90 (27.6)
T _3_ (≥ 6001 bath)	152 (32.5)	59 (41.6)	93 (28.5)
Levels of physical activity, n(%)			
Low and moderate	219 (46.8)	45 (31.7)	174 (53.4)
Vigorous	249 (53.2)	97 (68.3)	152 (46.6)
Smoking status, n (%)			
No	374 (79.9)	51 (35.9)	323 (99.0)
Yes	94 (20.1)	91 (64.1)	3 (1.0)
Family history of DM/HT/dyslipidaemia, n (%)			
No	254 (54.3)	90 (63.4)	164 (50.3)
Yes	214 (45.8)	52 (36.6)	162 (49.7)
Medication use ^†^, n (%)			
No	430 (91.9)	134 (94.4)	296 (90.8)
Yes	38 (8.1)	8 (5.6)	30 (9.2)
MetS, n (%)			
No	285 (60.9)	92 (64.8)	193 (59.2)
Yes	183 (39.1)	50 (35.2)	133 (40.8)
Dietary intake, mean ± SD			
Total energy intake (kcal/d)	2102.2 ± 586.7	2278.3 ± 567.2	2067.5 ± 585.5 **
Carbohydrate (g/d)	396.2 ± 111.6	426.3 ± 114.2	390.3 ± 110.5 **
Protein (g/d)	61.1 ± 21.1	64.9 ± 19.8	60.3 ± 21.3 **
Fat (g/d)	28.2 ± 21.3	30.2 ± 19.3	27.8 ± 21.7 **
Fibre (g/d)	9.1 ± 7.3	8.6 ± 4.0	9.2 ± 7.8
Cholesterol (mg/d)	191.6 ± 120.4	208.7 ± 123.4	188.2 ± 119.8
Sugar (g/d)	48.7 ± 38.2	52.0 ± 28.5	48.0 ± 39.9

DM: diabetes mellitus; HT: hypertension ^†^Medication use for treating type 2 diabetes mellitus or high blood pressure or dyslipidemia * p-value < 0.0001 by using the Student’s *t*-test; ** p-value < 0.001 by using the Mann–Whitney *U* test.

The distribution of the components of MetS within the group of participants without MetS and those with MetS are given in Table 2. The proportion of individual components of MetS varies between the two groups as given in the table. Of the 248 participants with abdominal obesity, 62.1% falls into the group of MetS, but a considerable proportion of those with this component, 37.9%, were not in the MetS group. Likewise, the proportion of participants without MetS but with FBG above the cut-off point was18.6%; for low HDL-C, it was 45.6%; for high blood pressure was 32.4%; and for high triglyceride was 31.3%.

**Table 2.** Proportion of components of metabolic syndrome (MetS) in the groups of study participants without and with MetS.

Components of MetS	Total N = 468	Non-MetS N = 285	MetS N = 183
Abdominal obesity (%)	248	94 (37.9)	154 (62.1)
High blood pressure (%)	210	68 (32.4)	142 (67.6)
High fasting blood glucose (%)	70	13 (18.6)	57 (81.4)
High triglyceride (%)	195	61 (31.3)	134 (68.7)
Low HDL-C (%)	294	134 (45.6)	160 (54.5)

Abdominal obesity (waist circumference ≥ 90 cm in men or ≥ 80 cm in women); high blood pressure (SBP ≥ 130; DBP ≥ 85 mmHg or HT treatment); high fasting blood glucose (FBG ≥100 mg/dL or DM treatment); high triglyceride (TG ≥ 150 mg/dL) low HDL-C (HDL-C <40 mg/dL in male; <50 mg/dL in women

Two dietary patterns, named traditional and mixed, were identified by using factor analysis ( Table 3). The two patterns together explained 26.9% of the total variance. The variance for the mixed pattern was 14.2% and the variance for the traditional pattern was 12.7%. The mixed pattern was characterized by the consumption of a variety of foods consisting of fruits, noodles and bread, milk and yogurt, soybean and soybean products, vegetables, eggs, sweet beverages, bakery and snacks, processed meat, legumes and nuts, seafood, and white rice. The traditional pattern included a high intake of sticky rice and animal source foods consisting of internal organs of animals, freshwater animals (frogs, pond snails, and small shrimps), poultry, processed meat (Thai sausage, fermented pork sausage, hot dog sausage, and pork cracking), insects, red meat, fish, and seafood as well as energy drinks. Alcohol was not included into both the mixed pattern and traditional pattern because of a very low factor loading.

**Table 3.** Food groups with factor loading for two major dietary patterns.

Food groups	Mixed pattern	Traditional pattern
1. Fruits	0.575	-
2. Noodles and bread	0.553	-
3.Milk and yogurt	0.542	-
4. Soybean and soybean products	0.524	-
5. Vegetables	0.480	-
6. Eggs	0.455	-
7.Sweet beverages	0.434	-
8. Bakery and snacks	0.378	-
9. Processed meat	0.376	0.453
10. Legumes and nuts	0.370	-
11. Seafood	0.372	0.304
12. White rice	0.362	–0.467
13. Sticky rice	–0.323	0.603
14. Internal organs of animals	-	0.566
15. Poultry	-	0.499
16. Freshwater animals (frogs, pond snails, small shrimps)	-	0.477
17. Insects	-	0.427
18. Energy drink	-	0.411
19. Red meat	-	0.366
20. Fish	-	0.311
21. Alcohol	-	-
Total of variance explained (%)	14.2	12.7

Factor loading less than 0.30 are not displayed.

The distribution of age, sex, MetS and the five components of MetS according to tertile scores of the two dietary patterns are presented in Table 4. The proportions of men and women were significantly different according to tertiles of each dietary pattern. Participants in the highest tertile of mixed pattern were women more than men. Participants with MetS did not differ in the highest tertile of each dietary pattern score comparing to the lowest tertile score. The proportions of those participants in the second and third tertile score who were found to consume the traditional dietary pattern were significantly associated with high triglyceride levels in comparison with those falling into the first tertile. A reversed feature evolved for low HDL-C in those preferring the traditional pattern. The proportion within the tertiles of the mixed pattern did not differ significantly as far as the five components of MetS were concerned.

**Table 4.** The distribution of age, sex, metabolic syndrome (MetS) and its components according to tertiles of dietary pattern scores.

	Mixed pattern			p-value	Traditional pattern			p-value
	T1	T2	T3		T1	T2	T3	
	(n =156)	(n =156)	(n =156)		(n =156)	(n =156)	(n =156)	
Age, n (%)				0.03				0.01
35–49 years	63 (40.4)	78 (50.0)	86 (55.1)		64 (41.0)	73 (46.8)	90 (57.7)	
≥ 50 years	93 (59.6)	78 (50.0)	70 (44.9)		92 (59.0)	83 (53.2)	66 (42.3)	
Sex, n (%)				<0.001				<0.001
Men Women	68 (43.6) 88 (56.4)	42 (26.9) 114(73.1)	32 (20.5) 124(79.5)		21 (13.5) 135(86.5)	50 (32.1) 106(67.9)	71 (45.5) 85 (54.5)	
MetS,n (%) No Yes	92 (59.0) 64 (41.0)	91 (58.3) 65 (41.7)	102(65.4) 54 (34.6)	0.369	95 (60.9) 61 (39.1)	92 (59.0) 64 (41.0)	98 (62.8) 58 (37.2)	0.795
Components of MetS, n (%)								
Abdominal obesity	86 (55.1)	88 (56.4)	74 (47.4)	0.229	91 (58.3)	79 (50.6)	78 (50.0)	0.260
High BP	76 (48.7)	75 (48.1)	59 (37.8)	0.100	64 (41.0)	75 (48.1)	71 (45.5)	0.448
High FBG	23 (14.7)	23 (14.7)	24 (15.4)	0.100	27 (17.3)	22 (14.1)	21 (13.5)	0.594
High TG	71 (45.5)	65 (41.7)	59 (37.8)	0.387	54 (34.6)	68 (43.6)	73 (46.8)	**0.04**
Low HDL-C	94 (60.3)	102(65.4)	98 (62.8)	0.645	109(69.9)	97 (62.3)	88 (56.4)	**0.04**

T: tertiles of dietary pattern scores, BP: blood pressure; FBG: fasting blood glucose; TG: triglyceride; HDL-C: high density lipoprotein cholesterol

Table 5 shows the relationship of the mixed and traditional dietary patterns with MetS and each component of MetS after applying a multiple logistic regression, controlling for other covariates. The lowest tertile of each dietary pattern was considered as reference. By using this approach, neither the mixed nor the traditional pattern was associated with MetS. Comparing the lowest tertile score of the mixed and traditional patterns with the highest tertile score, the mixed pattern was inversely associated with abdominal obesity (adjusted OR=0.49, 95% CI: 0.30–0.81). Increasing consumption of the traditional pattern was likely to be associated with high TG (adjusted OR= 1.74, 95% CI: 1.10–2.88).

**Table 5.** Adjusted odds ratios (95% CI) for metabolic syndrome (MetS) and each component of MetS according to tertiles of each dietary pattern score.

	MetS	Abdominal obesity	High BP	High FBG	High TG	Low HDL-C
Mixed pattern						
T _1_ (n =156)	1.00	1.00	1.00	1.00	1.00	1.00
T _2_ (n =156)	1.02 (0.64 – 1.64)	0.79 (0.48 – 1.30)	1.21 (0.75 – 1.95)	1.17 (0.59 – 2.32)	0.99 (0.62 – 1.56)	1.09 (0.67 – 1.75)
T _3_ (n =156)	0.72 (0.45 – 1.18)	0. 49 (0.30 – 0.81)	0.75 (0.46 – 1.23)	1.08 (0.54 – 2.17)	0.88 (0.55 – 1.42)	0.93 (0.57 – 1.50)
*p* for trend ^*^	0.340	**0.01**	0.393	0.422	0.743	0.606
Traditional pattern						
T _1_ (n =156)	1.00	1.00	1.00	1.00	1.00	1.00
T _2_ (n =156)	1.36 (0.82 – 2.21)	1.05 (0.64 – 1.70)	1.48 (0.91 – 2.41)	0.98 (0.48 – 1.97)	1.46 (0.90 – 2.36)	0.86 (0.52 – 1.41)
T _3_ (n =156)	1.34 (0.81 – 2.22)	1.34 (0.81 – 2.26)	1.36 (0.81 – 2.27)	1.01 (0.48 – 2.14)	1.74 (1.10 – 2.88)	0.77 (0.46 – 1.27)
*p* for trend ^*^	0.229	0.353	0.381	0.986	**0.02**	0.374

BP: blood pressure;FBG: fasting blood glucose; TG: triglyceride; HDL-C: high density lipoprotein cholesterol; T: tertiles of dietary pattern scores

^*^All models were adjusted for age, sex, smoking status, level of physical activity, and medication use.

Raw data gathered from the questionnaire Click here for additional data file. Copyright: © 2019 Chupanit P et al. 2019 Data associated with the article are available under the terms of the Creative Commons Zero "No rights reserved" data waiver (CC0 1.0 Public domain dedication).

## Discussion

Two prominent dietary patterns of this cross-sectional study among northeast Thai people in the context of nutritional transition were generated by using the factor analysis, namely, the mixed pattern and the traditional pattern. A positive association was found between the traditional pattern and high TG, whereas a negative association was observed between the mixed pattern and abdominal obesity.

The traditional pattern as assessed through this study showed that a distinctive pattern still follows a traditional way of food intake such as eating more sticky rice and animal source foods. The consumption of freshwater animals (frogs, pond snails, and small shrimps), internal organs of animals, and insects of various kinds have long been recognised as ingredients of the northeast diet ^4^. Following the classification of a diet as traditional, sticky rice as a staple food can be rightly considered a traditional food. Previous studies reported that an ethnic-specific diet, characterized by high consumption of rice, legumes, and vegetables, was positively related to MetS and its components (low HDL-C) ^31^, while another study did not find the association with MetS and its components ^32^. The traditional pattern in this study, although not associated with MetS, nevertheless was significantly associated with high triglycerides. This finding might be explained by the intake of high amounts of sticky rice in the traditional pattern. Sticky rice (Oryzaglutinosa) has a high glycemic index ^33^ and stimulates high serum triglyceride levels ^34^. Recently, the Fourth Thai National Health Examination Survey found that “carbohydrate dietary pattern” characterized by consumption of sticky rice, fermented fish, chili paste and bamboo shoots was associated with the risk of MetS and its components such as high TG and low HDL-C ^17^. A study from the Korean National Health and Nutrition Examination Survey also showed that the high intake of rice-oriented pattern was significantly correlated with a higher prevalence of high TG and low HDL-C ^35^. Traditional patterns in this study not only included a high intake of sticky rice but also a high intake of animal source foods. Meat is an important source of total visible and invisible fat intake, particularly saturated fat. Several studies showed that dietary saturated fat consumption is associated with the risk of CVD due to increasing plasma lipid and lipoprotein ^36, 37^. However, the intake of several animal source foods in this pattern, comprising insects, freshwater animals (frogs, pond snails, small shrimps) and fish, are good sources of protein and are typically low in fat. This may explain the lack of association between the traditional pattern and the MetS, but this pattern was related to high TG.

The mixed pattern was characterized by a consumption of a wide variety of food items long known to be part of the Thai cuisine (such as white rice, fruits, and vegetables), mixed with food items marketed by the food industry (such as noodles of various brands and bread, milk and yogurt, bakery and snacks, sweet beverages, and soybean and soybean products). A previous study indicated that a dietary pattern consisting of a variety of foods (such as grains and starches, vegetables and fruits, fish and seafood, meat, poultry, eggs, legumes, and dairy products) was inversely associated with the incidence of MetS among Korean adults ^38^. Fruits, milk and yogurt, soybean and soybean products and vegetables, major foods characterising the mixed pattern, seem to constitute a beneficial diet ^39, 40^. Fruits and vegetables constitute a high-fibre diet, which is associated with a decreased risk of obesity ^40^, potentially accounting for some of the association between the mixed pattern and decreased abdominal obesity in this study. The meta-analysis of randomized controlled trials (RCTs) showed that the consumption of dairy products may have a modest benefit in facilitating weight loss in short-term or energy-restricted RCTs ^39^.

As evident from Table 2, a rather high proportion of participants had one or two components of MetS (such as low HDL-C, abdominal obesity, high BP, and high TG), but they were not included in the MetS group. Thus, MetS as such could not be statistically associated with two dietary patterns, but some components of MetS, significant statistically, could be linked to dietary patterns. High triglycerides were significantly linked to the consumption of the traditional pattern, whereas participants with no abdominal obesity related to the mixed pattern. Interpreting the statistics with caution, the mixed pattern appears to be more beneficial for health than the traditional pattern. Fruits and vegetables have long been constituents of the overall Thai dietary intake, but white rice is still the staple food throughout the country ^4^ and should not be considered unhealthy. What appears to be less beneficial in the traditional pattern is the intake of glutinous rice (Oryzaglutinosa) with a high glycemic index ^33^, which stimulates high serum triglyceride levels ^36^. Hypothetically, elevated triglyceride levels as linked to the traditional pattern here indicates the risk for consumers of this pattern to develop T2DM, and the mixed pattern might to a certain extent prevent consumers of this dietary intake from acquiring T2DM. High triglyceride blood levels have been found to be independent risk factors pointing toward the development of diabetes ^41– 43^. A promising approach for further studies would be to include metabolic variables closely linked to T2DM, such as insulin resistance, found to occur in hypertriglyceridemia and seem to be a risk factor for T2DM ^44^.

Some limitations of this study should be considered. Dietary patterns by using factor analysis involves subjective decisions for instant the consolidation of food items into food groups, the number of dietary patterns to be retained and the identified dietary patterns could be different in case of the subjects due to different ethnicity or culture ^14^. The other limitation of the cross-sectional study is not optimal for assessing causal relationship because the investigator measures the outcome and the exposures at a single period time.

## Conclusion

Two different dietary patterns could be recognized; the traditional- and the mixed pattern. These dietary patterns were not found to be associated with MetS. Adherence to the traditional pattern among the northeast Thai adults, in the context of nutrition transition, was associated with high triglyceride levels and seemed to be less beneficial for the mixed pattern, which was inversely related to abdominal obesity. Our findings show that the consumption of a variety of foods may be beneficial for health outcomes. Further studies on a larger sample population, as part of a prospective study, will provide important insights into the associations of dietary patterns and the metabolic syndrome.

## Data availability

The data referenced by this article are under copyright with the following copyright statement: Copyright: © 2019 Chupanit P et al.

Data associated with the article are available under the terms of the Creative Commons Zero "No rights reserved" data waiver (CC0 1.0 Public domain dedication). 

Dataset 1: Raw data gathered from the questionnaire 10.5256/f1000research.15075.d207245 ^45^

## Supplementary materials

Supplementary File 1: Questionnaire used to interview participants. The questionnaire comprises of 4 part (1) General information, (2) Health information, (3) Global Physical Activity Questionnaire (GPAQ), (4) Food intake assessment by a semi-quantitative food frequency questionnaire.

.

Supplementary File 2: Table S1 - Food lists of 20 food groups in the study.
