# Supplementary material for: Dietary patterns and their association with the components of metabolic syndrome: A cross-sectional study of adults from northeast Thailand
Source: F1000Res. 2019 Apr 3;7:905. Originally published 2018 Jun 25. [Version 2] doi: 10.12688/f1000research.15075.2 (PMC6480943; doi:10.12688/f1000research.15075.2)
Supplement: Supplementary file 2 [file f1000research-7-20291-s0001.tgz › 0f07039b-9b9c-4cd8-a848-15314d749d40.docx]

**ID Number**

**Questionnaire for “Dietary patterns and their association with the components of metabolic syndrome: A northeast Thai experience”**

This objective of this questionnaires are to investigate dietary patterns and to determine the relationship between dietary patterns and MetS and its components. Please answer the questions frankly in your opinion. Your answers are going to benefit for this study. All of answers will be confidential.

This questionnaire has 4 parts which include;

Part 1 General information

Part 2 Health information

Part 3 Global Physical Activity Questionnaire (GPAQ)

Part 4 Food intake assessment

I hope to have a good cooperation from you. Thank you.

Miss Pornpimon Chupanit

Doctoral student

Faculty of public health

Khon Kaen University

**Introduction:** Kindly tick ✓mark the answer in the given column

| **Part 1 General information** | **Researcher** |
| --- | --- |
| 1. Sex ( ) 1. male ( ) 2. female 2. Age.....................years 3. Marital Status   ( ) 1. Single ( ) 2. Married ( ) 3. Widowed ( ) 4. Divorced   1. Education levels   ( ) 1. Uneducated  ( ) 2. Elementary school  ( ) 3. High school  ( ) 4. College/bachelor's degree   1. Occupation   ( ) 1. Not working  ( ) 2. Farmer  ( ) 3. Employee  ( ) 4. Government officer  ( ) 5. Retail / Business  ( ) 6. Other............................................................................................   1. Income..............................................Bath/month 2. Smoking status   ( ) 1. No ( ) 2.Yes | sex ( )  age ( )  status ( )  edu ( )  occ ( )  inc ( )  smoking  ( ) |
| **Part 2 Health information** | **Researcher** |
| 1. Do you have any health problems by medical diagnosis? (you can choose more than one answer)   ( ) 1. Diabetes Mellitus  ( ) 2. Hypertension  ( ) 3. Hyperlipidemia  ( ) 4. Cardiovascular diseases  ( ) 5. Other (pleases to specify)..............................................................................   1. Do you have to use medical drugs for treatment?   ( ) 1. No  ( ) 2. Yes (pleases to specify)....................................................................................................  …………………………………………………………………………   1. Do you have family history of a first-degree relative with diabetes mellitus or hypertension or dyslipidaemia?   ( ) 1. No  ( ) 2. Yes (pleases to specify)............................................................... | Dx( )  Px ( )  FH ( ) |

**Part 3** Global Physical Activity Questionnaire (GPAQ)

**Introduction:**

The Global Physical Activity Questionnaire was developed by WHO for physical activity surveillance in countries. It collects information on physical activity participation in three domains (activity at work, travel to and from places, recreational activities) and sedentary behavior.

Next I am going to ask you about the time you spend doing different types of physical activity in a typical week. Please answer these questions even if you do not consider yourself to be a physically active person. Think first about the time you spend doing work. Think of work as the things that you have to do such as paid or unpaid work, study/training, household chores, harvesting food/crops, fishing or hunting for food, seeking employment. [Insert other examples if needed]. In answering the following questions 'vigorous-intensity activities' are activities that require hard physical effort and cause large increases in breathing or heart rate, 'moderate-intensity activities' are activities that require moderate physical effort and cause small increases in breathing or heart rate.

| **Questions** | | **Response** | **Code** |
| --- | --- | --- | --- |
| - 1. Activity at work | | | |
| 1. | Does your work involve vigorous-intensity activity that causes large increases in breathing or heart rate like [carrying or lifting heavy loads, digging or construction work] for at least 10 minutes continuously? For example lifting or carrying heavy loads, digging, construction work, etc. | - Yes - No   (If No, go to P4) | P1 |
| 2. | In a typical week, on how many days do you do vigorous intensity activities as part of your work? | Number of days  ...................  per week | P2 |
| 3. | How much time do you spend doing vigorous-intensity activities at work on a typical day? | Hours : minutes  □□ **:** □□ | P3  (a-b) |
| 4. | Does your work involve moderate-intensity activity that causes small increases in breathing or heart rate such as brisk walking [or carrying light loads] for at least 10 minutes continuously? | - Yes - No   (If No, go to P7) | P4 |
| **Questions** | | **Response** | **Code** |
| 5. | In a typical week, on how many days do you do moderate intensity activities as part of your work? | Number of days  ...................  per week | P5 |
| 6. | How much time do you spend doing moderate-intensity activities at work on a typical day? | Hours : minutes  □□ **:** □□ | P6  (a-b) |
| - 1. **Travel to and from places** | | | |
| The next questions exclude the physical activities at work that you have already mentioned. Now I would like to ask you about the usual way you travel to and from places. For example to work, for shopping, to market, to place of worship. | | | |
| 7. | Do you walk or use a bicycle (pedal cycle) for at least 10 minutes continuously to get to and from places? | - Yes - No   (If No, go to P10) | P7 |
| 8. | In a typical week, on how many days do you walk or bicycle for at least 10 minutes continuously to get to and from places? | Number of days  ...................  per week | P8 |
| 9. | How much time do you spend walking or bicycling for travel on a typical day? | Hours : minutes  □□ **:** □□ | P9  (a-b) |
| - 1. **Recreational activities** | | | |
| The next questions exclude the work and transport activities that you have already mentioned. Now I would like to ask you about sports, fitness and recreational activities (leisure). | | | |
| 10. | Do you do any vigorous-intensity sports, fitness or recreational (leisure) activities that cause large increases in breathing or heart rate like [running or football,] for at least 10 minutes continuously? | - Yes - No   (If No, go to P13) | P10 |
| 11. | In a typical week, on how many days do you do vigorous intensity sports, fitness or recreational (leisure) activities? | Number of days  ...................  per week | P11 |
| 12. | How much time do you spend doing vigorous-intensity sports, fitness or recreational activities on a typical day? | Hours : minutes  □□ **:** □□ | P12  (a-b) |
| **Questions** | | **Response** | **Code** |
| 13. | Do you do any moderate-intensity sports, fitness or recreational (leisure) activities that causes a small increase in breathing or heart rate such as brisk walking,(cycling, swimming, volleyball)for at least 10 minutes continuously? | - Yes - No   (If No, go to P16) | P13 |
| 14. | In a typical week, on how many days do you do moderate-intensity sports, fitness or recreational (leisure) activities? | Number of days  ...................  per week | P14 |
| 15. | How much time do you spend doing moderate-intensity sports, fitness or recreational (leisure) activities on a typical day? | Hours : minutes  □□ **:** □□ | P15  (a-b) |
| - 1. **Sedentary behavior** | | | |
| The following question is about sitting or reclining at work, at home, getting to and from places, or with friends including time spent [sitting at a desk, sitting with friends, travelling in car, bus, train, reading, playing cards or watching television], but do not include time spent sleeping. | | | |
| 16. | How much time do you usually spend sitting or reclining on a typical day? | Hours : minutes  □□ **:** □□ | P16 |

**Part 4** Food intake assessment by a semi-quantitative food frequency questionnaire

**Introduction:** Please fill the number of times and amount of food in the space.

| **Food items** | **Your serving** | **How often did you eat food items within the last month?** | | | | **Code** |
| --- | --- | --- | --- | --- | --- | --- |
|  |  | **times/ day** | **times/ week** | **times/ month** | **Never or less than once/month** |  |
| **Rice and rice products, and cereals** | | | | | | |
| 1. White rice |  |  |  |  |  |  |
| 2. sticky rice |  |  |  |  |  |  |
| 3. unpolished rice |  |  |  |  |  |  |
| 4. Corn |  |  |  |  |  |  |
| 5. Sweet potato |  |  |  |  |  |  |
| 6. Thai rice noodle (*Khanom chin*) |  |  |  |  |  |  |
| 7. Noodle |  |  |  |  |  |  |
| 8. Instant noodle |  |  |  |  |  |  |
| 9. Vermicelli |  |  |  |  |  |  |
| 10. spaghetti and macaroni |  |  |  |  |  |  |
| 11. White bread |  |  |  |  |  |  |
| 12. Cracker and biscuits |  |  |  |  |  |  |
| **Nut and legumes and soybean products** | | | | | | |
| 13. Peanut |  |  |  |  |  |  |
| 14. Cashew nut |  |  |  |  |  |  |
| 15. Sun Flower seed |  |  |  |  |  |  |
| 16. Watermelon seed |  |  |  |  |  |  |
| 17. Pumpkin seed |  |  |  |  |  |  |
| 18. Sesames |  |  |  |  |  |  |
| 19. Kidney bean |  |  |  |  |  |  |
| 20. Black bean |  |  |  |  |  |  |
| 21. Soybean |  |  |  |  |  |  |
| 22. Tofu |  |  |  |  |  |  |
| 23. Soybean milk |  |  |  |  |  |  |
| **Food items** | **Your serving** | **How often did you eat food items within the last month?** | | | | **Code** |
|  |  | **times/ day** | **times/ week** | **times/ month** | **Never or less than once/month** |  |
| **Vegetables** | | | | | | |
| Green leafy vegetables (Please specify);  (24)………………………  (25)………………………  (26)……………………… |  |  |  |  |  |  |
| White leafy vegetable  (Please specify);  (27)………………………  (28)………………………  (29)……………………… |  |  |  |  |  |  |
| Root vegetables  Please specify;  (30)………………………  (31)………………………  (32)……………………… |  |  |  |  |  |  |
| 33. Mushroom |  |  |  |  |  |  |
| 34. Tomato |  |  |  |  |  |  |
| 35. Cucumber |  |  |  |  |  |  |
| 36. Bamboo |  |  |  |  |  |  |
| 37. Other………………… |  |  |  |  |  |  |
| **Fruits** | | | | | | |
| 38. Banana |  |  |  |  |  |  |
| 39. Dragon fruit |  |  |  |  |  |  |
| 40. cantaloupe |  |  |  |  |  |  |
| 41. Rambutan |  |  |  |  |  |  |
| 42. Rose apple |  |  |  |  |  |  |
| 43. Apple |  |  |  |  |  |  |
| 44. Mango |  |  |  |  |  |  |
| **Food items** | **Your serving** | **How often did you eat food items within the last month?** | | | | **Code** |
|  |  | **times/ day** | **times/ week** | **times/ month** | **Never or less than once/month** |  |
| 45. Guava |  |  |  |  |  |  |
| 46. Pineapple |  |  |  |  |  |  |
| 47. Ripe papaya |  |  |  |  |  |  |
| 48. Orange |  |  |  |  |  |  |
| 49. Mangos teen |  |  |  |  |  |  |
| 50. Watermelon |  |  |  |  |  |  |
| 51. Other………………… |  |  |  |  |  |  |
| **Meat and meat products** | | | | | | |
| 52. Pork |  |  |  |  |  |  |
| 53. Beef |  |  |  |  |  |  |
| 54. Chicken |  |  |  |  |  |  |
| 55. Duck |  |  |  |  |  |  |
| 56. Eggs (Please specify)………………….. |  |  |  |  |  |  |
| 57. Thai sausage or Sai krok Isan |  |  |  |  |  |  |
| 58. Fermented pork sausage |  |  |  |  |  |  |
| 59. Hot dog sausages |  |  |  |  |  |  |
| 60. Pork cracking (fried pork skin) |  |  |  |  |  |  |
| Internal organs of animal (Please specify);  (61)………………………  (62)………………………  (63)……………………… |  |  |  |  |  |  |
| **Food items** | **Your serving** | **How often did you eat food items within the last month?** | | | | **Code** |
|  |  | **times/ day** | **times/ week** | **times/ month** | **Never or less than once/month** |  |
| Fish (fresh water fish)  Please specify;  (64)………………………  (65)………………………  (66)……………………… |  |  |  |  |  |  |
| 67. Mackerel fish |  |  |  |  |  |  |
| **Freshwater animals** | | | | | | |
| 68. Frogs |  |  |  |  |  |  |
| 69. Pond snails |  |  |  |  |  |  |
| 70 Small shrimps |  |  |  |  |  |  |
| **Seafood** | | | | | | |
| 71. Shrimp |  |  |  |  |  |  |
| 72. Squid |  |  |  |  |  |  |
| 73. Crab |  |  |  |  |  |  |
| 74. Shell |  |  |  |  |  |  |
| **Insects** | | | | | | |
| 75. Grasshopper |  |  |  |  |  |  |
| 76. Bamboo worms |  |  |  |  |  |  |
| 77. Silkworms |  |  |  |  |  |  |
| 78. Other (Please specify)……………………………………………. |  |  |  |  |  |  |
| **Milk and milk products** | | | | | | |
| 79. Milk (Please specify)……………………………………………. |  |  |  |  |  |  |
| **Food items** | **Your serving** | **How often did you eat food items within the last month?** | | | | **Code** |
|  |  | **times/ day** | **times/ week** | **times/ month** | **Never or less than once/month** |  |
| 80. Yogurt (Please specify)……………………………………………. |  |  |  |  |  |  |
| 81. Fermented milk |  |  |  |  |  |  |
| **Bakery and snacks** | | | | | | |
| 82. Cake |  |  |  |  |  |  |
| 83. Cookies |  |  |  |  |  |  |
| 84. Donut |  |  |  |  |  |  |
| 85. Chinese donut  or Chinese bread stick |  |  |  |  |  |  |
| 86. Snack (Please specify)……………………………………………. |  |  |  |  |  |  |
| 88. Other……………… |  |  |  |  |  |  |
| **Sweet beverages** | | | | | | |
| 88. Soft drink (Please specify)………………… |  |  |  |  |  |  |
| 89. Hot coffee (Please specify);  ( ) black coffee  ( ) with milk and sugar |  |  |  |  |  |  |
| 90. Iced coffee |  |  |  |  |  |  |
| 91. Iced tea |  |  |  |  |  |  |
| 92. Iced tea with milk |  |  |  |  |  |  |
| 93. Iced chocolate/ Cocoa |  |  |  |  |  |  |
| 94. Milkshake |  |  |  |  |  |  |
| 95. Fruit smoothies (Please specify)………………… |  |  |  |  |  |  |
| **Food items** | **Your serving** | **How often did you eat food items within the last month?** | | | | **Code** |
|  |  | **times/ day** | **times/ week** | **times/ month** | **Never or less than once/month** |  |
| 96. Fruit juice with sugar (Please specify)………….. |  |  |  |  |  |  |
| 97. Processed juice (Please specify)………………….. |  |  |  |  |  |  |
| 98. Energy drink (Please specify)………………….. |  |  |  |  |  |  |
| 99. Other………………… |  |  |  |  |  |  |
| **Alcohol** | | | | | | |
| 100. Beer |  |  |  |  |  |  |
| 101. Whiskey |  |  |  |  |  |  |
| 102. Rice whisky |  |  |  |  |  |  |
| 103. Brandy |  |  |  |  |  |  |
| 104. Other……………….. |  |  |  |  |  |  |

********Thank you for your cooperation********
