# Supplementary material for: Dietary patterns and their association with the components of metabolic syndrome: A cross-sectional study of adults from northeast Thailand
Source: F1000Res. 2019 Apr 3;7:905. Originally published 2018 Jun 25. [Version 2] doi: 10.12688/f1000research.15075.2 (PMC6480943; doi:10.12688/f1000research.15075.2)
Supplement: Supplementary file 3 [file f1000research-7-20291-s0002.tgz › c08f49de-6e7b-4ff8-bc44-b4532e7d7bab.docx]

**Table S1**

**Food lists of 20 food groups in the study**

| **Food Groups** | **Food list** |
| --- | --- |
| 1. White rice | Polished rice |
| 2. Sticky rice | Sticky rice |
| 3. Noodles and bread | White bread, sandwich, cracker, Chinese noodle, Thai rice noodle (*Khanom chin*), instant noodles, vermicelli, spaghetti, and macaroni |
| 4. Fish | Fish |
| 5. Poultry | Chicken, duck |
| 6. Red meat | Pork, beef |
| 7. Processed meat | Thai sausage or Sai krok Isan, fermented pork sausage, hot dog sausage, and pork cracking (fried pork skin) |
| 8. Vegetables | Asparagus, baby corn, bean sprouts, broccoli, cauliflower, cabbage, Chinese cabbage, carrot, cucumber, pumpkin, tomato, egg plant, bamboo shoot, mushroom, yard long bean, fresh papaya, sponge gourd, Chinese kale, lettuce, cassia leaves, basil leave, Thai water morning glory. |
| 9. Fruits | Tangerine orange, apple, mango, watermelon, banana, ripe papaya , guava, cantaloupe, pineapple, or seasonal fruits |
| 10. Milk and yogurt | Milk, Fermented milk, and yogurt |
| 11. Soybean and soybean products | Soy bean, tofu, and soy milk |
| 12. Egg | Egg |
| 13. Sweetened drinks | Coffee or tea with milk and sugar, carbonated beverages, soft drink, Iced chocolate or cocoa, milkshake, fruit smoothies, and so on |
| 14. Bakery and snacks | Cake, cookies, biscuit, and snacks |
| 15. Legumes and nuts | Sesames, watermelon seed, sunflower seed, pumpkin seed, peanut, mung bean, kidney bean, black bean, cashew nut |
| 16. Seafood | Shrimp, squid, crab, shell |
| 17. Internal organs of animals | Hearts, livers, and kidneys of pork or chicken |
| 18. Freshwater animals | Frogs, pond snails, small shrimps |
| 19. Insects | Cricket, grasshopper, bamboo worms, silkworms, and so on |
| 20. Energy drink | Energy drinks (trade name) such as Red Bull, M-150, and so on |
| 21. Alcohol | Beer, whisky, rice whisky, brandy, and so on |
