# Supplementary material for: Dietary patterns and their association with the components of metabolic syndrome: A cross-sectional study of adults from northeast Thailand
Source: F1000Res. 2019 Apr 3;7:905. Originally published 2018 Jun 25. [Version 2] doi: 10.12688/f1000research.15075.2 (PMC6480943; doi:10.12688/f1000research.15075.2)
Supplement: Peer review history

# Peer review: Reviewer response for version 2

**DOI:** 10.5256/f1000research.20291.r46679

**Published:** 2019-04-17

**Authors:** Nipa Rojroongwasinkul

**Affiliations:** 1 Institute of Nutrition, Mahidol University, Nakhon Pathom, Thailand

Thank you for your revision and clarification according to my comments.

I have read this submission. I believe that I have an appropriate level of expertise to confirm that it is of an acceptable scientific standard.

# Peer review: Reviewer response for version 1

**DOI:** 10.5256/f1000research.16416.r36423

**Published:** 2018-09-19

**Authors:** Sakda Pruenglampoo

**Affiliations:** 1 Research Institute for Health Sciences, Chiang Mai University, Chiang Mai, Thailand

It  may be concluded as the following:  

 1. Clear research objectives.

 2. The study design is appropriate for the objectives. However, it might be replaced by “Analytical cross sectional study design .

 3. The criteria which used in this study for outcome variables were suitable. However it should add some more details about the accuracy of the methods/instruments for measuring  anthropometric and biochemical measurements  and also the components of the metabolic syndrome.

 4 .The methods used for food intake assessment (Semi-FFQ, the picture of food sizes and kitchen equipment and INMUCAL-N version 2) sound reasonable for the study.

 5. Factor analysis used to distinguish the dietary pattern of the subjects has an advantage that it can reduce the number of variables , by combing two or more variables into single factor. However, it may have some limitations which the authors of this study have mentioned already.

 6.The methods of analysis and the statistical methods employed were suitable for the types of variables (nominal versus ordinal versus continuous) in this study.

 7.Regarding discussion part, it might be useful for the readers if the authors mention in briefly about possible sources of bias which might be occurred and how to control or to decrease the effect of the bias in the study.  

 8. In conclusion part, sample population in a prospective study should include all part of Thailand (north, north- eastern, central and south).

 9. Other comments for minor changes: (See PDF)

 9.1 Abstract:  A cross-sectional study should be changed to An analytical cross-sectional study.

 9.2 Introduction: Check grammar for writing through the chapter of introduction.

 9.3 Methods:  Put the meaning of STROBE in parentheses (The Strengthening the Reporting of Observational Studies in Epidemiology) and check grammar for writing through the chapter of methods.

 9.4 Results: Table 3 should have foot note to explain that factor loading less than 0.30 were not displayed and the factor loading shown in the table did not consider the signs of ±). Table 4 should have foot note to state the statistical methods for testing P value.

 9.5 Discussion: Check grammar for writing through the chapter of discussion.

 9.6 Conclusion: Check grammar for writing through the chapter of conclusion.

 9.7 References: Check format and completeness of the references.

I have read this submission. I believe that I have an appropriate level of expertise to confirm that it is of an acceptable scientific standard.

# Peer review: Reviewer response for version 1

**DOI:** 10.5256/f1000research.16416.r35562

**Published:** 2018-07-17

**Authors:** Nipa Rojroongwasinkul

**Affiliations:** 1 Institute of Nutrition, Mahidol University, Nakhon Pathom, Thailand

In "Abstract", session "Methods", the study was conducted among 468 healthy adults but some participants in study are not healthy, please write only 468 adults.

 In Table 1, focus at dietary intake, the result show the statistical significant difference of total energy intake between men and women.  Do you test the difference of the other nutrients intake such as carbohydrate, protein, fat, fiber, cholesterol, and sugar between men and women?  According to the distribution of these nutrients are skewed (not normal distribution), using Student’s t-test may not be appropriate test. For sure, testing normality of these nutrients, if the nutrient is not normal distribution the appropriate statistical testing is Mann-Whitney U Test (nonparametric statistic).

 Please show the prevalence of MetS separate by sex.

 The mixed pattern was characterized by the consumption of a variety of foods (factor loading >0.3) consisting of

1. Fruits (F=0.575 )
2. noodles and bread (F=0.553)
3. milk and yogurt (F=0.542)
4. soybean and soybean products (F=0.524)
5. vegetables (F=0.48)
6. eggs (F=0.455)
7. sweet beverages (F=0.434)
8. bakery and snacks (F=0.378)
9. processed meat (F=0.376)
10. legumes and nuts (F=0.37)
11. seafood (F=0.372)
12. white rice (F=0.362)

The traditional pattern included a high intake of rice (sticky & white rice) and animal source foods (factor loading >0.3) consisting of

1. sticky rice (F=0.603)
2. internal organs of animals (F=0.566)
3. poultry (F=0.499)
4. freshwater animals (frogs, pond snails, small shrimps) (F=0.477)
5. white rice (F= -0.467)
6. processed meat (Thai sausage, fermented pork sausage, hot dog sausage, and pork cracking) (F=0.453)
7. insects (F=0.427)
8. energy drinks (F=0.411)
9. red meat (F=0.366)
10. fish (F=0.311)
11. seafood (F=0.304)

The 2 pattern that authors suggested, in each pattern some variable are shown in both pattern (processed meat) and the variables in each pattern are not clear cut.

 I checked from your data and found that the participants in your study who consumed both sticky rice and white rice are 85% (398 from 468) so the dietary pattern in this study is not follow the traditional way of food intake that eating more sticky rice but they consumed both rice.

 From theory of factor analysis, the greater the loading, the more the variable is a pure measure of the factor. Comrey and Lee (1992) suggest that loadings in excess of .71 (50% overlapping variance) are considered excellent, .63 (40% overlapping variance) are considered very good, .55 (30% overlapping variance) are considered good, .45 (20% overlapping variance) are considered fair, and .32 (10% overlapping variance) are considered poor. [Cited in Tabachnick BG and Fidell LS. Using multivariate statistics. 6 ^th^ ed. New Jersey: Pearson Education, Inc.: 2013.]

 If we decided to use a loading of .45 (20% variance overlap between variable and factor). With the use of the .45 cut, we will get the variables in each factor are as follow:

**Factor 1: Healthier pattern**

 Fruits (F=0.575 )
 noodles and bread (F=0.553) 
milk and yogurt (F=0.542)
4. soybean and soybean products (F=0.524) 
 vegetables (F=0.48)
 eggs (F=0.455)

**Factor 2: Rice and meat pattern**

1. sticky rice (F=0.603)
2.  internal organs of animals (F=0.566)
3.  poultry (F=0.499)
4.  freshwater animals (frogs, pond snails, small shrimps) (F=0.477) 
5.  white rice (F= -0.467)
6.   processed meat (Thai sausage, fermented pork sausage, hot dog sausage, and pork cracking) (F=0.453)

Please consider to assign the name of the new factors I just try to assign the name but the name may be not appropriate.

 From the new factors that we got, please re-run the analysis and discuss the results accordingly.

I have read this submission. I believe that I have an appropriate level of expertise to confirm that it is of an acceptable scientific standard, however I have significant reservations, as outlined above.

# Response

**Published:** 2019-03-06

**Authors:** Pornpimon Chupanit

**Affiliations:** Thailand

1) In "Abstract", session "Methods", the study was conducted among 468 healthy adults but some participants in study are not healthy, please write only 468 adults.

**answer:** We had already revised.

 2) In Table 1, focus at dietary intake, the result show the statistical significant difference of total energy intake between men and women.  Do you test the difference of the other nutrients intake such as carbohydrate, protein, fat, fiber, cholesterol, and sugar between men and women?  According to the distribution of these nutrients are skewed (not normal distribution), using Student’s t-test may not be appropriate test. For sure, testing normality of these nutrients, if the nutrient is not normal distribution the appropriate statistical testing is Mann-Whitney U Test (nonparametric statistic).

**answer:** As suggested, the difference between groups has been tested now also by non-parametric statistics.We used the Mann-Whitney U Test for total energy carbohydrate protein and fat (showed in Table 1).

 3) Please show the prevalence of MetS separate by sex.

**answer: **We added the prevalence of MetSin Table 1.

 4) The 2 pattern that authors suggested, in each pattern some variable are shown in both pattern (processed meat) and the variables in each pattern are not clear cut.

 I checked from your data and found that the participants in your study who consumed both sticky rice and white rice are 85% (398 from 468) so the dietary pattern in this study is not follow the traditional way of food intake that eating more sticky rice but they consumed both rice.

 From theory of factor analysis, the greater the loading, the more the variable is a pure measure of the factor. Comrey and Lee (1992) suggest that loadings in excess of .71 (50% overlapping variance) are considered excellent, .63 (40% overlapping variance) are considered very good, .55 (30% overlapping variance) are considered good, .45 (20% overlapping variance) are considered fair, and .32 (10% overlapping variance) are considered poor. [Cited in Tabachnick BG and Fidell LS. Using multivariate statistics. 6 ^th^ ed. New Jersey: Pearson Education, Inc.: 2013.]

 If we decided to use a loading of .45 (20% variance overlap between variable and factor).

**answer: **In the international literature for dietary pattern studies this loading factor is frequently used and seems to be appropriate ^1-6^.So for instance Castro MA. et al. reported that to use ≥ 0.25 for factor loading was an acceptable to look into dietary patterns, so factor loading > 0.3 as use in our study should be appropriate. In a normal life situation it is unlikely that the majority of groups of the ordinary population sticks to a very fixed diet, especially not if the objective of the study was to test the dietary intake in the situation of the ‘nutritional transition’ which is mentioned in the abstract, the introduction and the discussion. Higher loading factors will ‘squeeze’ the data into ‘unnatural fixed groups’ representing those refusing to or not being influenced by the ‘transition’. Transition means that the majority of those tested might not stick on a daily basis to a very fixed dietary intake. One and the other food items will appear in both groups but with different loading factors as for instance for sticky rice and white rice (see table 3). It is mention that the majority of participants (85%) consumed both sticky rice and white rice. It is argued by the referee that the dietary pattern in this study doesn’t follow the traditional way of food intake in that that both kinds of rice is consumed. In Fact this result should be expected in the situation of a transition of dietary intake. The loading factor for sticky rice consumed for the here so called ‘traditional diet’ with a 0.6 exceeds the loading factor for sticky rice for the mixed group with -0.323 by far. A similar result was achieved for white rice where the traditional group had a loading factor of -0.467 much lower that the mixed group with 0.362.  So it can be concluded that the the traditional groups still have a strong tendency to follow the traditional way of consumption but already started to alternate between both kinds of rice.

 1.    Aekplakorn W, Satheannoppakao W, Putwatana P, Taneepanichskul S, Kessomboon P, Chongsuvivatwong V, et al. Dietary pattern and metabolic syndrome in thai adults. J Nutr Metab. 2015;2015:468759.

 2.    Naja F, Nasreddine L, Itani L, Adra N, Sibai AM, Hwalla N. Association between dietary patterns and the risk of metabolic syndrome among Lebanese adults. Eur J Nutr. 2013;52(1):97-105.

 3.    Panagiotakos DB, Pitsavos C, Skoumas Y, Stefanadis C. The Association between Food Patterns and the Metabolic Syndrome Using Principal Components Analysis: The ATTICA Study. Journal of the American Dietetic Association. 2007;107(6):979-87.

 4.    Suliga E, Kozieł D, Cieśla E, Głuszek S. Association between dietary patterns and metabolic syndrome in individuals with normal weight: a cross-sectional study. Nutrition Journal. 2015;14(1):55.

 5.    Woo HD, Shin A, Kim J. Dietary patterns of Korean adults and the prevalence of metabolic syndrome: a cross-sectional study. PLoS One. 2014;9(11):e111593.

 6.    Castro MA, Baltar VT, Selem SS, Marchioni DM, Fisberg RM. Empirically derived dietary patterns: interpretability and construct validity according to different factor rotation methods. Cad Saude Publica. 2015;31(2):298-310.
